# Green and sustainable chitosan–gum Arabic nanocomposites as efficient anticorrosive coatings for mild steel in saline media

**DOI:** 10.1038/s41598-022-17386-7

**Published:** 2022-08-01

**Authors:** Sherief A. Al Kiey, Mohamed S. Hasanin, Fakiha El-Taib Heakal

**Affiliations:** 1 Electrochemistry and Corrosion Department, National Research Centre (NRC), Dokki, 12622 Cairo Egypt; 2 Cellulose and Paper Department, National Research Centre (NRC), Dokki, 12622 Cairo Egypt; 3 Chemistry Department, Faculty of Science, Cairo University, Giza, 12613 Egypt

**Keywords:** Corrosion, Nanoscale materials

## Abstract

The application of green and sustainable anticorrosive coatings is becoming of upsurge interest for the protection of metallic materials in aggressive environments. Herein, a stable crystalline chitosan/gum Arabic composite (CGAC) nanopowder was successfully synthesized and characterized by various methods. The CGAC nanopowder with different doses (25, 50, 100, and 200 ppm) was used to coat mild steel samples and examined its anticorrosion ability in 3.5 wt.% NaCl solution using gravimetric, electrochemical measurements, and surface characterization techniques. All methods yielded consistent results revealing that nanocomposite coatings can impart good anticorrosive properties to the steel substrate. The obtained protection efficiency was enhanced with increasing CGAC dose in the applied surface layer achieving 96.6% for the 200 ppm-coating. SEM and AFM surface morphologies of uncoated and coated samples after the inundation in the saline solution showed that CGAC coating can block the active corrosive sites on the steel surface, and prevent the aggressive Cl^-^ ions from attacking the metallic substrate. The water droplet contact angle gave further support as it increased from 50.7° for the pristine uncoated surface to 101.2° for the coated one. The current research demonstrates a promising natural and reliable nanocomposite coating for protecting mild steel structures in the marine environment.

## Introduction

Green and efficient coatings are among the key approaches for protecting the appearance, strength, performance, and functionality of most metallic structures from the attack of the environment. Therefore, the development of advanced functional and smart anti-corrosive coatings in many technological applications is currently a major focus of the scientific academy. Chitosan (Ch) is a linear copolymer comprising β-(1,4)-2-amido-2-deoxy-D-glucan (glucosamine) and β-(1,4)-2-acetamido-2-deoxy-D-glucan (N-acetylglucosamine) that may be synthesized from chitin via partial alkaline deacetylation. Chitin is the second most prevalent polysaccharide in nature, after cellulose, and is widely distributed around the world, generally extracted from the shells of crustaceans, and the exoskeleton of many arthropods. Polysaccharides are the largest category of biopolymers, mainly derived from plants, animals, fungi, and bacteria^1,2^. The features of polysaccharide biopolymers are compatible with worldwide requirements, especially towards the environment^3–5^. Because of their natural origin, these natural polymers are biodegradable, nontoxic, highly reactive with multiple adsorption sites, and a wide range of specifications^6,7^. When chitosan is dissolved in a dilute acetic acidic solution, the amine groups are protonated, and the resulting positive charges endow the macromolecule polyelectrolyte-like traits. Biocompatibility, antibacterial activity, biodegradability, and exceptional excellent film-forming ability are only a few of its distinctive physicochemical characteristics that have enticed the attention of many researchers. These intriguing physicochemical properties among others have piqued scientific and industrial interest in a variety of fields, including biotechnology, pharmaceutics, biomedicine, packaging, wastewater treatment, cosmetics, and food science^8–12^. Due to its unique properties, including high film-forming ability, superior adherence to metallic surfaces, and versatility associated with the ease of chemical functionalization, chitosan, and its composites can be a viable option for applications as a protective coating barrier against corrosion of metallic substrates as for copper-based and aluminium-based alloys^13,14^. Also, Gebhardt et al.^15^ have characterized the behavior of electrophoretic chitosan coatings on stainless steel under physiological conditions. Meanwhile, John et al.^16^ have used the sol–gel dip coating approach for studying the corrosion inhibition of mild steel by chitosan/TiO_2_ nanocomposites coatings in acid solutions. Likewise, chitosan and some of its derivatives can be used as inhibitors for corrosion of carbon steel^17^ and stainless steel^18^ in 3.5% NaCl. However, the single components alone are not effective enough against corrosive media (acid, alkaline or neutral) and may have many drawbacks in the large-scale usage, where the solubility, as well as stability, would be of paramount interest^19–21^. Consequently, the use of polysaccharides composites is more required in the industry to obtain promising results^22–25^.

Gum Arabic (GA), also known as Arabic gum, is a natural branched-chain complex polysaccharide derived from exudates of stems and branches of Acacia Senegal or of related Acacia species. The chemical composition of GA can vary with its source, the age of trees from which it was obtained, climatic conditions, and soil environment. Gum Arabic is either neutral or slightly acidic, edible and soluble in water. It is commonly used in industries for film forming, encapsulation, and as a food additive due to its unique combination of excellent emulsifying properties and low solution viscosity^26^. GA contains the *l*-arabinose, *l*-rhamnose, and *d*-glucuronic acid. Its backbone is composed of 1,3-linked β-d-galactopyranosyl units and the side chains are composed of two to five 1,3-linked β-d-galactopyranosyl units, joined to the main chain by 1,6-linkages^27,28^. The literature survey indicated that there are voluminous research works dealing with the use of GA as anticorrosive materials for some metal/electrolyte systems^29–32^. In addition, Verma and Quraishi^33^ have recently reviewed the assortment of literature on GA as an environmentally sustainable alternative to classical organic corrosion inhibitors. As for Ch/GA composite so far, it has only been exploited as a useful approach to delay the ripening process and reducing the deterioration of fruits during cold storage^34^. But to the best of our knowledge, information is lacking relevant to the use of Ch/GA composite as anticorrosive material. Therefore, in this work, Ch/GA nanocomposite was synthesized from natural Ch and GA biopolymers using a simple ultrasonic-assisted process. The fabricated nanocomposite powder was physically analyzed by several characterization techniques. For the first time, we are reporting the application of the obtained Ch/GA nanocomposite as anticorrosion green coatings for mild steel in aerated stagnant saline solution. Assessment of the anticorrosion performance was achieved using gravimetric immersion test, as well as electrochemical PDP and EIS techniques. Furthermore, the corroded bare and coated mild steel surfaces were scrutinized employing the scanning electron microscopy (SEM), energy dispersive X-ray spectroscopy (EDX), atomic force microscopy (AFM), and water droplet contact angle measurements.

## Experimental part

### Materials

Chitosan (Ch) used in this study were purchased from Sigma Aldrich (St. Louis, molecular weight 650,000, viscosity 275.9 cps, and degree of deacetylation 85.5%). Gum Arabic (GA) was also purchased from Sigma Aldrich (USA), CAS number 26,077–0, Acacia powder [9000-01-5], with an average molecular weight 380,000 and its appearance color is white to faint beige.

### Nanocomposite preparation

**Figure 1:**
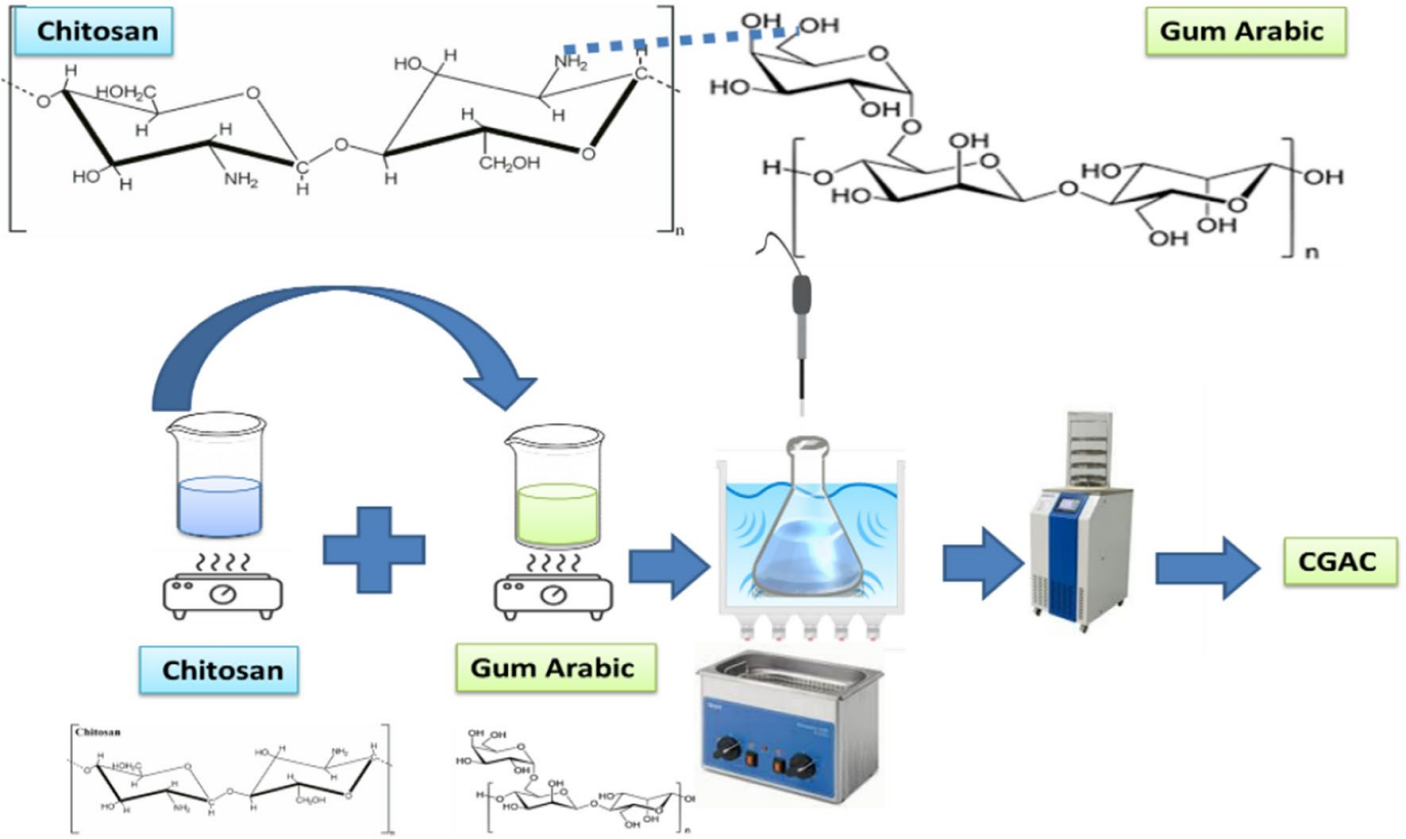
An image for the preparation process of CGAC nanopowder.

One mole of repeating unit of both Ch and GA were vigorously stirred in100 mL of 1% (v/v) acetic acid individually for 2 h at 70 °C. Next, each solution was sonicated using an ultra sonicator prop for 15 min in an ice bath. The above two solutions were then mixed together and sonicated at 60 °C for 2 h in 1000 W ultrasonic water bath. At the same time, 0.1 M NaOH was added till neutralization. The collected solution was then lyophilized and keep dry in the dark for further investigations and uses. The specific preparation process is as illustrated in Fig. 1.

### Physicochemical characterization

Information relevant to the composition and structure of the prepared inhibitor powder were collected using FT-IR spectrometer (Nicolet Impact-400 FT-IR spectrophotometer) in the range of 400–4000 cm^−1^. Also, the X-ray diffraction (XRD) patterns of samples was investigated on a Diano X-ray diffractometer using a CuKα radiation source energized at 45 kV and a Philips X-ray diffractometer (PW 1930 generator, PW 1820 goniometer) with CuKα radiation source (λ = 0.15418 nm). The XRD patterns were recorded in a diffraction angle range of 2*θ* from 10° to 80° in the reflection mode. The topographical study was carried out using scanning electron microscopy (SEM) provided with energy dispersive electron spectroscopy unit (EDX) (JSM 6360 L V, JEOL/Noran). Cross section images of the surface coating were also examined using SEM to assess the quality and thickness of the film. For the surface morphology imaging, different samples were recorded using an accelerating voltage of 10–15 kV. TEM model JEM2010, Japan, was used to investigate the particle size and morphology of the synthesized nanocomposite powder. The zeta potential of the composite was measured using NicompTM 380 ZLS size analyzer, USA. Laser light scattering was used at 18°. Thermogravimetric and differential thermogravimetric analyses (TGA and DTGA) of the CGAC and its two pure components were performed in the nitrogen atmosphere with heating rate of 10 °C/min using SDT Q600 thermal analyser, USA.

### Weight loss measurements

For corrosion rate determination by the chemical approach, mild steel coupons with dimensions of (20 mm × 20 mm × 3 mm) were obtained in the following chemical composition, (wt. %): 0.19% C, 0.05% Si, 0.94% Mn, 0.009% P, 0.004% S, 0.014% Ni, 0.009% Cr, 0.034% Al, 0.016% V, 0.003% Ti, 0.022% Cu, and the rest Fe. The mild steel substrates were abraded using finer emery papers (600–1500 grit), then rinsed with distilled water and dried in the open air. Each three steel substrate coupons were then dipped at the same time for 1 h in the chitosan-gum Arabic composite (CGAC)/1% acetic acid solution with the specified concentration of 25, 50, 100, or 200 ppm, then pulled up slowly from the solution. The steel substrates with the coated layers were finally dried for 2 h at 80 °C and weighted. After weighing the pieces, they were immersed in a beaker containing 100 mL of the corrosive 3.5 wt.% NaCl solution for a definite time period of 24 h. After that, they were removed from the bath, cleaned several times with deionized water, and reweighted. The corrosion rate (*C.R.*, µg cm^-2^ h^-1^) and the protection efficiency (*η*_*w*_%) of mild steel coated chitosan–gum Arabic nanocomposite (CGAC) were calculated using Eqs. (1) and (2), respectively^35,36^:

1 $$C.R. = \frac{{W_{b} - W_{a} }}{S \times t}$$

2 $$\eta_{w} \% = (1 - \frac{{\left( {C.R.} \right)_{a} }}{{\left( {C.R.} \right)_{b} }} \times 100$$

where *W*_b_ and *W*_a_ are the sample average masses before and after the exposure time (*t*, h), and *S* is the total surface area of the sample in cm^2^.

### Electrochemical measurements

In order to demonstrate the corrosion behavior of the different samples, electrochemical impedance spectroscopy (EIS) and potentiodynamic polarization (PDP) measurements were performed using Voltalab 40 instrument and Voltamaster programme. The electrochemical testes for the coated and as-abraded steel samples were done in aerated 3.5 wt.% NaCl solution at room temperature (25 ± 1 °C) to simulate the actual application environment. The test solutions were prepared with analytical grade chemicals and doubly distilled water. A traditional three-electrode cell was used for the electrochemical measurements, encompassing mild steel sample as the working electrode, Ag/AgCl (sat KCl) as the reference electrode, and a large platinum sheet as the counter electrode. The working electrode substrate with a surface area of 1 cm^2^ was handled as described in the weight loss technique before each test. The perturbation signal amplitude used for the EIS technique was 10 mV peak to peak in the frequency domain 10^5^–10^–2^ Hz. The Tafel polarization curves were recorded at a scan rate of 0.5 mV s^-1^ in the potential range of –950 mV and –600 mV (vs. Ag/AgCl). Prior each electrochemical measurements, the mild steel electrode was left dipped into the test saline solution for 1 h to achieve a steady-state open-circuit potential condition. Each electrochemical experiment was carried out three times and average values of the similar results have been reported.

### Sample surface topology

The surface morphology of the bare and CGAC nanocomposite coated mild steel samples after 24 h immersion in 3.5 wt.% NaCl solution were examined using the QUANTA FEG 250 FE-SEM. AFM (Santa Barbara, CA, USA) was also used to measure the surface roughness and surface topography in nanoscale for the same two samples.

## Results and discussion

### Physicochemical characterization of the nanocomposite

#### FT-IR spectroscopy

**Figure 2:**
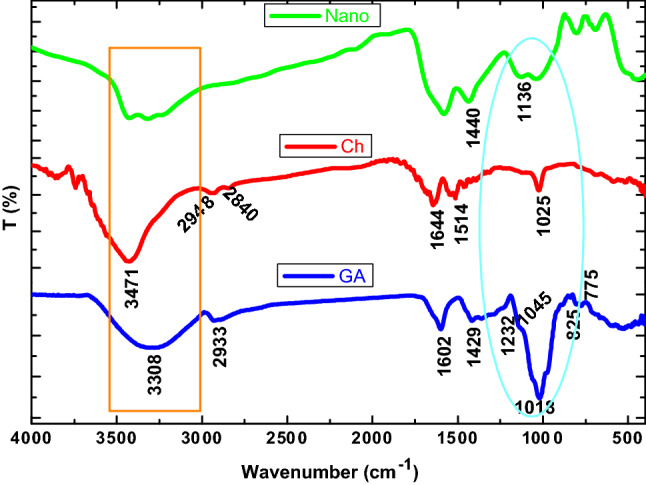
FT-IR of the prepared nanocomposite inhibitor and its two raw materials Ch and GA.

Fourier transform infrared spectroscopy (FT-IR) spectroscopy is an effective technique to identify the functional groups that may be present in different substances. Figure 2 shows the FT-IR spectra indicating the functional groups involved in the synthesized CGAC nanocomposite compared to those of the two pure polysaccharide polymers. As can be seen, chitosan (Ch) spectrum displays function group bands at: 3471, 2948, 2840, 1644, 1514, and 1025 cm^−1^ assigned, respectively, to the N–H stretching, symmetric –CH_3_ and asymmetric –CH_2_, C–H stretching, the C = O stretching (amide I), and –NH stretching (amide II), as well as the free amino group (–NH_2_) at C2 position of glucosamine, respectively. Also, FT-IR spectrum of gum Arabic (GA) exhibits significant bands representative of pure GA at: 3308, 2933, 1602, 1429, 1232, 1045, 1018, 825, and 775 cm^−1^ indicative, respectively, of O–H stretching vibration, C–H stretching of CH_2_ group, COOH stretching, uronic acid (COOH), NH bending vibration of the amide group, C–O–C of the amide (referred to protein fraction), and linkage of polysaccharide, galactose & mannose, and (1–4) & (1–6) linkages of galactose & mannose^37^. However, for CGAC nanopowder the OH group appeared as the broadest band in comparison with the raw materials. In addition, the band at 1440 cm^−1^ which refers to C–H bending of the methyl group is recorded as a sharp band due to interaction of the two components together. Moreover, the bands of C = C stretching and N–H bending are overlapped in a single band. This may be referred to the interaction of the protein region of GA with amino groups of the Ch. In this context, the C–O stretching band of primary alcohol appears at 1136 cm^−1^ as a sharp band, and the characteristic band of the polysaccharide’s linkage becomes broader. All these observations confirm well that interaction between GA and Ch is carried out at the intermolecular level.

#### XRD spectroscopy

**Figure 3:**
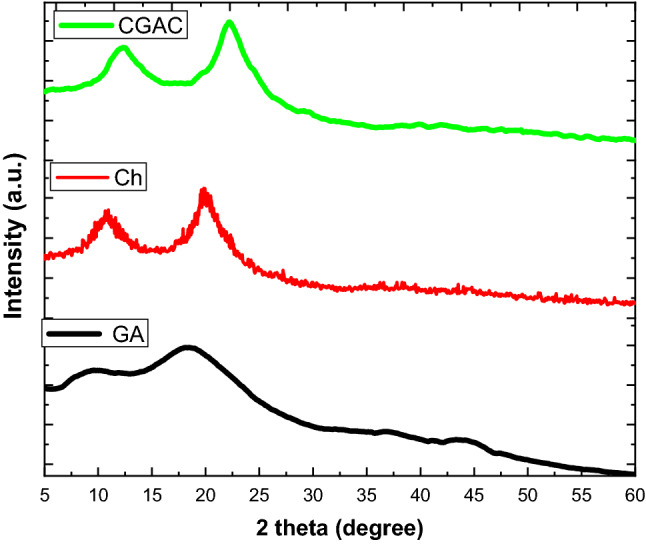
Crystallographic patterns of the prepared nanocomposite powder and its two raw materials.

Figure 3 illustrates the crystallographic patterns of the GA, Ch and CGAC nanopowder. The GA and Ch execute a polysaccharide behavior which consists of simple, repetitive sequences prefer to adopt helical conformations ^38^. Consequently, GA exhibits an amorphous spectrum with two intense diffraction peaks at 2*θ* = 8° and 18.8° which are signed to the amorphous structure of gum Arabic ^39,40^. Ch spectrum approves two hubs at intensities around 2*θ* of 11° and 21° referred to the conventional XRD pattern of the pristine chitosan ^41^. In view of the CGAC nanopowder, XRD pattern exhibits clear intensities that are located in near positions with moderate shifting, which may be referred to the 3D dimension of the nanocomposite (CGAC). Otherwise, the crystallinity of the CGAC nanopowder may be changed according to the peaks position, intensity and sharpness which refer to the mixed phase of amorphous and crystalline pattern.

#### Zeta potential

**Figure 4:**
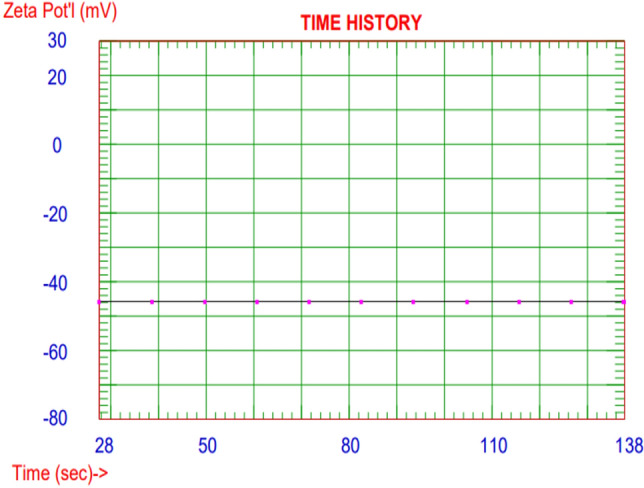
Zeta potential measurement of the prepared nanocomposite.

Zeta potential denotes the stability of a colloidal system and the net surface charge that its nanoparticles have, which is important for understanding its performance. Figure 4 represents the zeta potential chart of CGAC nanocomposite being recorded at a high potential value of about -45.78 mV. This result affirms that our prepared inhibitor is highly stable in its solution ^42^.

#### Morphological study

**Figure 5:**
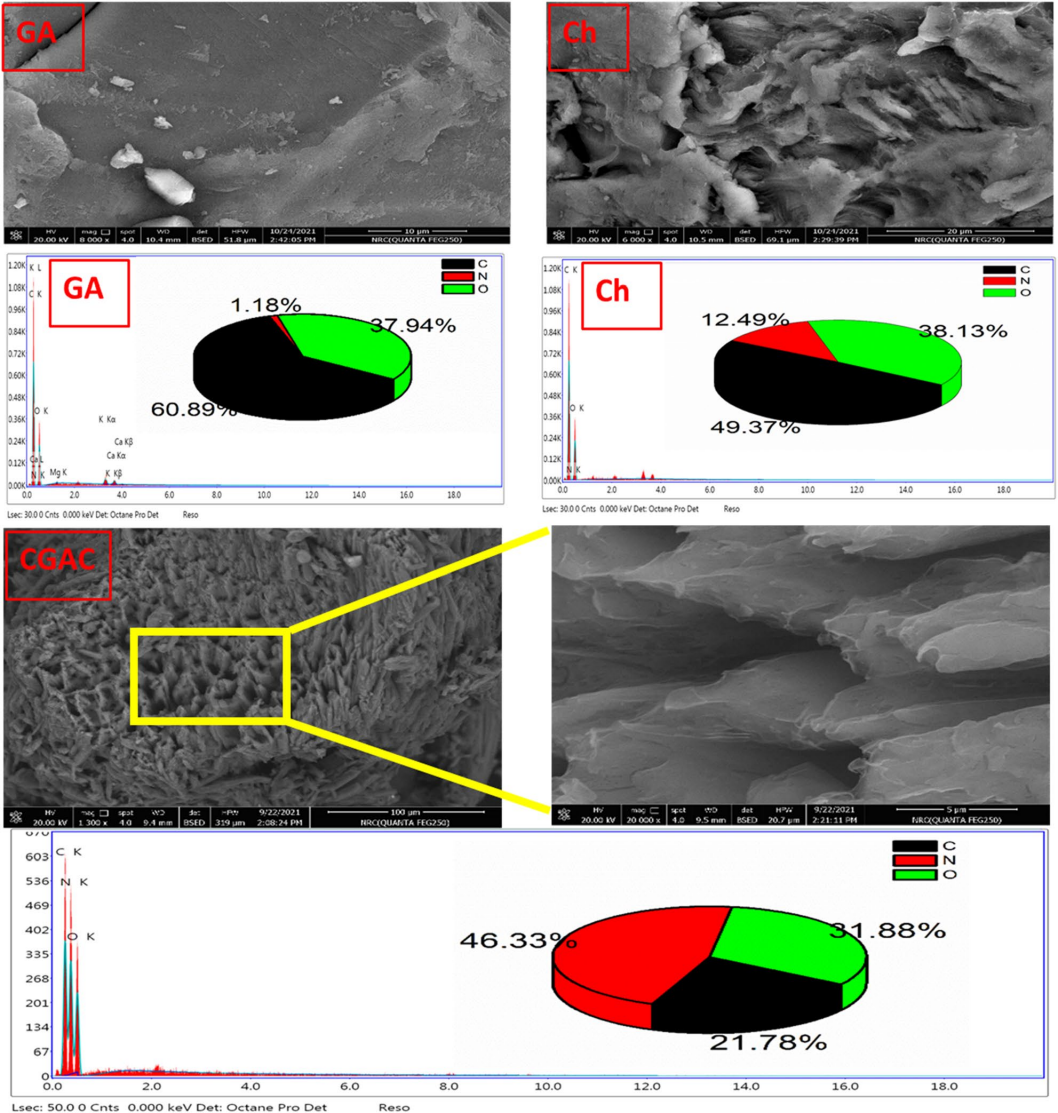
SEM images as well as EDX charts of the two raw materials (upper) and the CGAC nanopowder (lower).

EDX is a valuable tool for determining the characteristics of a new molecule because it can provide both qualitative and quantitative data. Figure 5 shows the surface morphologies of the two raw materials and CGAC nanopowder, as well as their EDX charts. The morphology of each raw material reveals a smooth surface with many folds without a specified surface appearance like nonfibrous polysaccharide polymers. Besides, EDX chart records the presence of carbon, oxygen and nitrogen in both raw materials with some ions involved in GA, like calcium, magnesium, and potassium as traces. The surface morphology appearance of Ch/GA nanocomposite looks as nanosheets in the low magnification image. In the meantime, the higher magnification image illustrates that layer structure being clear as a rough surface with intra-structure of pucker-like shape. Moreover, the EDX chart confirms that the elemental nanocomposite components including carbon, nitrogen, and oxygen. These results emphasize that the prepared nanocomposite is surely recorded in nanoscale with a unique surface structure.

#### TEM study

**Figure 6:**
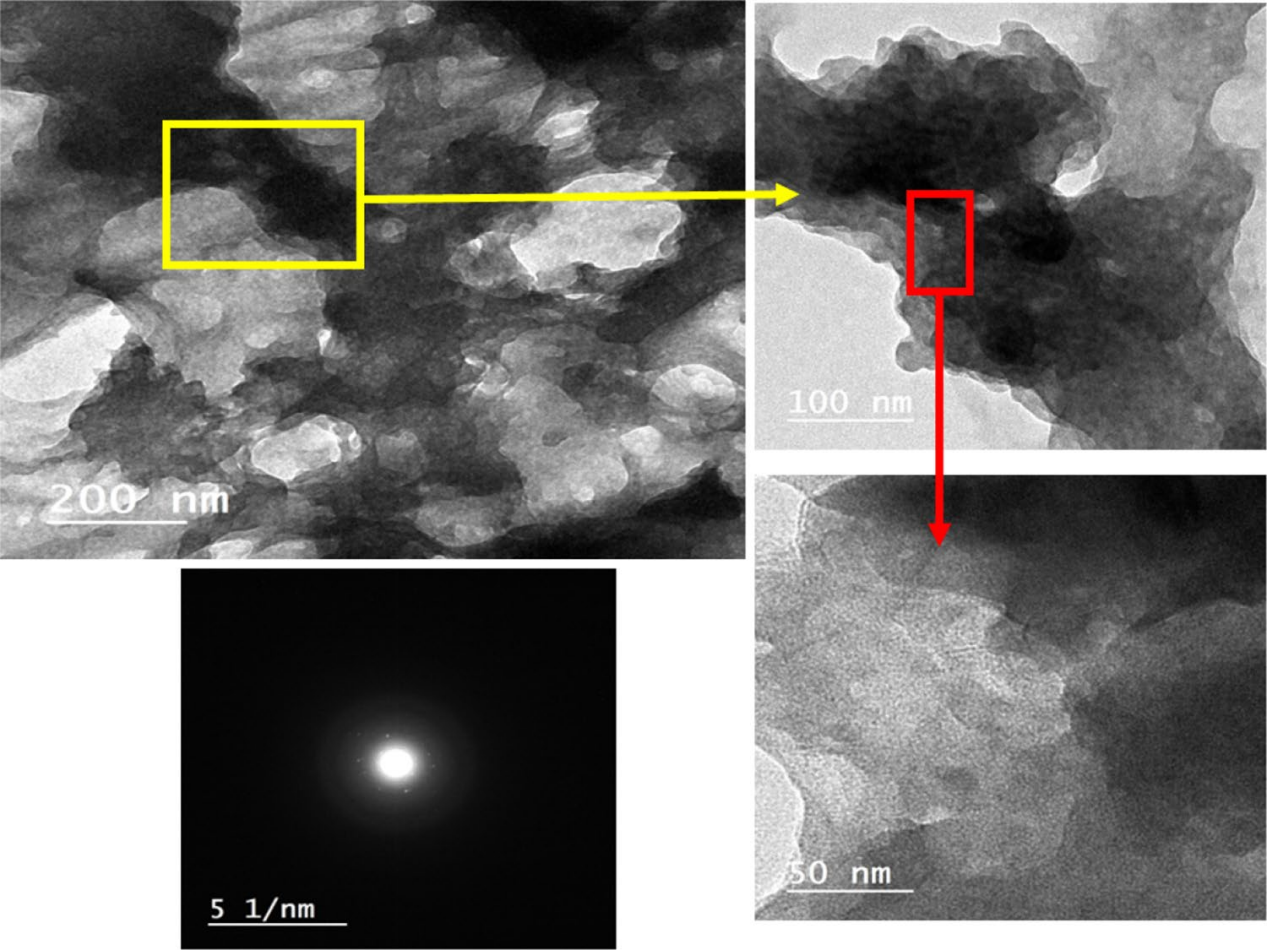
TEM images of Ch/GA nanocomposite at three different magnifications and its SAED pattern.

The intermolecular structure of the prepared nanocomposite was studied via TEM as shown in Fig. 6. The low magnification image indicates that the shape of the nanocomposite structure is mostly irregular circular nanosheets arranged on top of each other. Also, the higher magnification images were taken with two different magnification powers. The images confirm that the circular nanosheets are thin spheres in the nanosized range of about 84 nm. Moreover, the selected area electron diffraction (SAED) pattern of CGAC appears with a faint rings containing random dispersed spots that refereed to non-pure polycrystalline pattern mixed with an amorphous region of crystalline species related to material containing nano aggregations. This observation is supported by the XRD conclusion, as the CGAC was found to be a mixture of both crystalline and amorphous parts in the nanoscale dimension ^43^.

It is well established that, metallic corrosion is a mixed process involving two redox systems, oxidation partial reaction (metal corrosion) and reduction partial reactions ^30^. To prevent this unwanted process, it is essential to control both oxidation and reduction reactions. Organic coating as a physical barrier has been widely used for isolating the metallic structure from its surrounding. This would restrict the diffusion of corrosive ions through the pores and cracks on the substrate surface and it is important to use nanomaterials to perfectly fill those gaps. However, engineering the interface depends on the shapes and types of the nanoparticles. For our synthesized CGAC nanopowder, the TEM images revealed that it has a small nearly rounded shape, that would lead to a smooth surface formation and thus perfect protection when it is applied as a coating for the steel.

#### Thermal analysis

**Figure 7:**
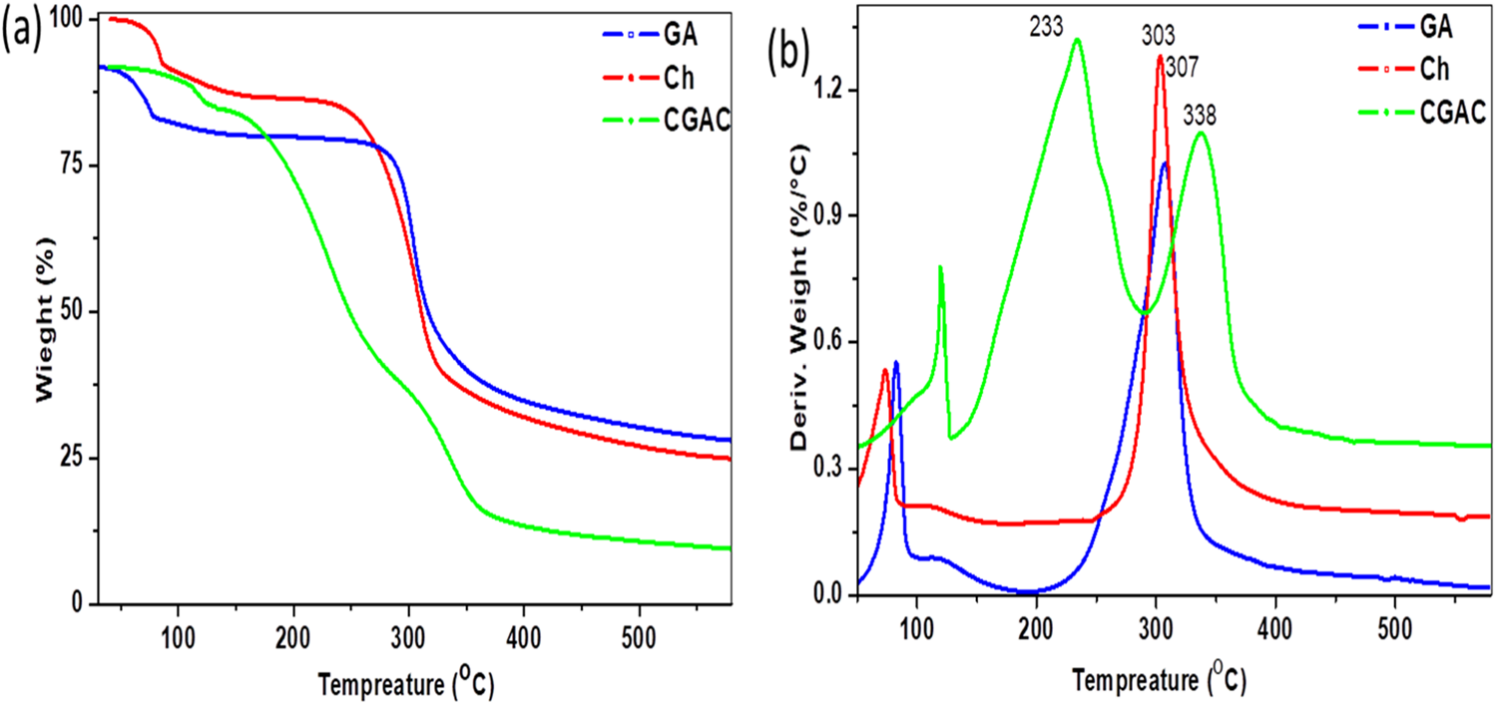
(**a**) TGA and (**b**) DTGA for CGAC nanopowder and its parent materials.

The thermal analyses shown in Fig. 7 include thermal gravimetric analysis (TGA) and differential thermal gravimetric analysis (DTGA) of GA, Ch and Ch/GA nanocomposite (CGAC). Thermal behavior can confirm the interaction between polymers by tracing the thermal behavior changes of the started materials and the products ^44^. GA exhibits a typical trait of a pure polysaccharide, where the thermal decomposition was carried out during a single band allocated at 307 °C with weight loss of about 47%. In this context, Ch performs a similar thermal characteristic to the GA, where the decomposition band was recoded as one step process at 303 ^o^C with weight loss of about 32%. In the meantime, the nanocomposite displays unique thermal behavior of two-stage decomposition with two peaks at 234 ^o^C and 338 °C with weight loss of about 39% and 74%, respectively. These results prove that the interaction between the two raw materials has been taken place and the produced CGAC nanopowder exhibits different thermal features. The phenomena is unique for the nanocomposite based polysaccharides, which usually happen due to polymer chain length reduction during the nanocomposite production process. The nanocomposite formation leads to shorten the polymer chains, as a consequence of the interaction between the two polymers active groups. Herein, it is obvious that the thermal behavior of the produced nanocomposite exhibit high thermal stability with a high weight loss.

### Anticorrosion performance of the nanocomposite

#### Gravimetric tests

**Figure 8:**
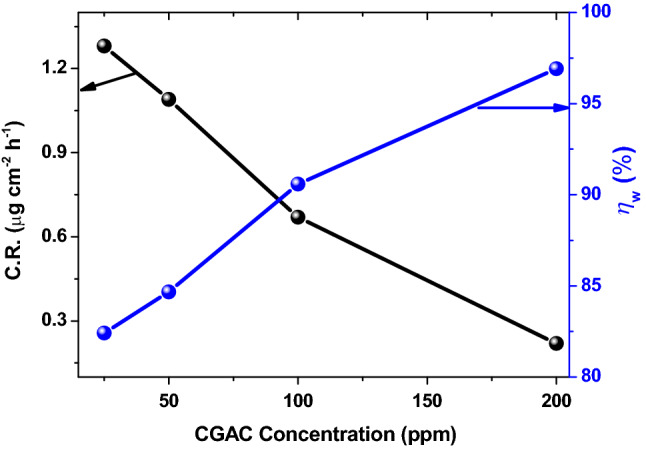
Dependence of the corrosion rate (*C.R.*) and protection efficiency (*ɳ*_*w*_%) obtained from the weight loss method, on the CGAC nanopowder dose in the coating.

The weight loss measurement was carried out to demonstrate the protection efficacy of CGAC thin coatings containing different concentrations from the nanocomposite powder on mild steel corrosion in neutral saline solution (3.5 wt.% NaCl). The corrosion data such as corrosion rate (*C.R*.) in µg cm^-2^ h^-1^ and protection efficiency (*ɳ*_*w*_%) as calculated from Eqs. (1) and (2), respectively, are presented in Fig. 8 as a function of the nanocomposite powder concentration in the coatings. As can be seen, the corrosion rates of coated mild steels are all lower than the uncoated bare sample and its value decreases significantly with increasing the dose of CGAC nanopowder in the coated layer, and subsequently *η*_*w*_% increases achieving a maximum value of 96.91% for the 200 ppm coating. The results manifest that CGAC establishes an excellent coating layer on mild steel that can effectively seal the active corrosion sites on the metal surface against chloride ions assault, thus decreasing its corrosion rate in the aerated aggressive saline solution ^45^. The barrier protection performance property is improved with increasing the nanocomposite dose in the coating. The data in Table 1 confirm a significant reduction in the corrosion rate (C.R.) of coated mild steel samples, that were left immersed 24 h in 3.5 wt.% NaCl solution, with the upsurge in the nanocomposite Ch/GA contents in the coating. In the meantime, the inhibitory efficiency (*ɳ*_w_%) promptly increases from about 82% at a dose of 25 ppm CGAC and reaching a maximum value of 96.91% at a dose of 200 ppm. The obtained results demonstrate the good performance of our proposed anticorrosive Ch/GA nanocomposite coatings in the saline environment.

**Table 1:** Weight loss corrosion rate (*C.R*.) and the protection efficiency (*ɳ*_w_%) of the different coated mild steel samples as measured in 3.5 wt.% NaCl solution after 24 h immersion.

CGAC dose in the coating	*C.R*. (µg cm^-2^ h^-1^)	*ɳ*_*w*_ (%)
Bare steel	7.11 ± 0.005	–
25 ppm	1.28 ± 0.004	82.41
50 ppm	1.09 ± 0.001	84.67
100 ppm	0.67 ± 0.003	90.58
200 ppm	0.22 ± 0.002	96.91

#### Electrochemical tests

To further gather more information regarding the feasibility of utilizing the synthesized chitosan/gum Arabic nanocomposite (CGAC) as anticorrosive green and sustainable coatings for mild steel in saline media, electrochemical corrosion investigations via potentiodynamic Tafel polarization plots and electrochemical impedance spectroscopy were exploited for bare uncoated mild steel and coated mild steel samples.

#### Potentiodynamic polarization measurements

**Figure 9:**
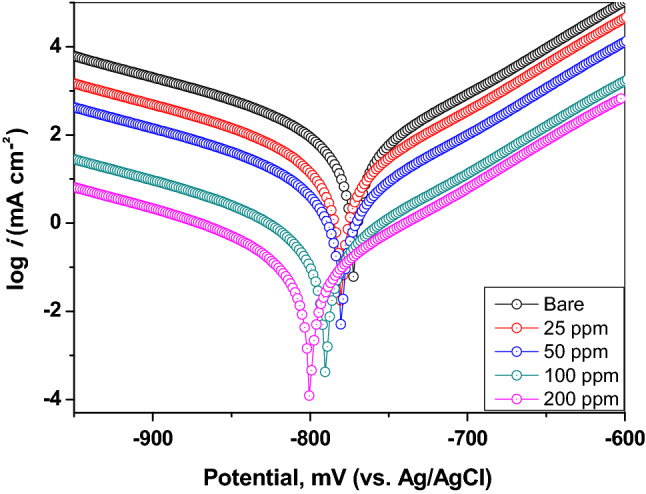
Potentiodynamic polarization curves of the bare and different CGAC coated mild steel samples in 3.5 wt.% NaCl solution at 25 °C.

PDP studies are mostly used to assess the corrosion resistance and the reliability of protective coatings ^46^. Figure 9 depicts typical potentiodynamic polarization curves of mild steel samples coated with a thin layer of CGAC containing different amounts from the nanocomposite powder, namely, 25, 50, 100, and 200 ppm, as well as the uncoated substrate being all measured in aerated saline solution. The Tafel plot (log *i* vs. *E*) was recorded for each sample over the potential range –1000 mV up to –600 mV (vs. Ag/AgCl) at 25 °C. The variation of the logarithm of current density with potential is predicted by the Tafel equations to be a straight line. By extrapolating the anodic and cathodic branches, electrochemical kinetic corrosion parameters could be derived from the polarization plots, such as anodic and cathodic Tafel slopes (*β*_a_ and *β*_c_), corrosion potential (*E*_corr_), and corrosion current density (*i*_corr_). The protection efficiency (*η*_*Tafel*_%) was also estimated, to evaluate the effectiveness of the Ch/GA nanocomposite coating in suppressing mild steel dissolution in saline environment, using the following equation ^47,48^:

3 $${\eta }_{Tafel}\%=\left(1-\frac{i}{{i}_{o}}\right)\times 100$$

where *i*_o_ and *i* are the values of the corrosion current densities obtained for the uncoated and coated samples, respectively. Figure 9 clearly demonstrates that the presence of Ch/GA nanocomposite coatings reduces the values of both anodic and cathodic current densities indicating a corresponding reduction in the anodic dissolution and cathodic reduction reactions. Accordingly, these nanocomposite coatings can be considered as mixed type inhibitors. Moreover, it is well established that for the corrosion process of mild steel in aerated neutral saline solution, the anodic reaction is mainly the released metal ions from the substrate surface into the solution (Fe → Fe^2+^  + 2e-). While the cathodic reaction is attributed to the oxygen and water molecules reduction (½O_2_ + H_2_O + 2e- → 2OH^-^). Notably, Fig. 9, reveals that for any Ch/GA coated sample ${i}_{corr}$ is of lower value than that for the bare steel surface. In fact, the obtained ${i}_{corr}$ value for the uncoated sample was found to be 53.2 µA cm^-2^ and decreases considerably for the coated mild steel surfaces until attaining a smaller value of 2.1 µA cm^-2^ for the 200 ppm CGAC coating. In the meantime, the calculated protection coefficient ${(\eta }_{Tafel}$%) was found to increase continuously with increasing the CGAC dose in the coated layer achieving a higher value of 96.05% for the 200 ppm coating, in a good coherence with ${\eta }_{w}$% value obtained from the weight loss method (96.91%).

Table 2 summarizes the estimated electrochemical corrosion parameters derived from the polarization curves as a function of CGAC powder dose in the coated layer. Triplicate determinations of the corrosion current density (*i*_corr_) values were made with the uncoated and with each of the coated samples. The corresponding average protection efficiency $\tt \tt \tt \it {(\eta }_{Tafel}\%)$ reported in Table 2 was found to have a standard deviation (*SD*) value in the range of 2.0 – 2.4%. The results reveal an obvious reduction in *i*_corr_ value on using increased Ch/GA composite concentration in the coated layer with a continuous shift in *E*_corr_ towards more negative potential values. According to Li et al. ^49^, if the difference in corrosion potential between the coated and bare electrode is greater than ± 85 mV, the inhibitor is either anodic or cathodic. In the present situation, the greatest displacement in *E*_corr_ value is only -43 mV indicating that CGAC nanocomposite in the coatings functions as a mixed type of inhibitor with a predominance cathodic barrier behavior. Besides, the shift in Tafel slope curves can be correlated to successful surface modification, which results in higher dispersion and filling efficiencies while avoiding aggregation, and the improved barrier protection is attributed to the steric spatial anchoring effect ^32^. The Ch/GA nanocomposite coatings can be considered as a barrier layer that can perfectly prevent corrosive species from reaching to the mild steel surface. Their good anticorrosive performance is mainly relevant to their effective sealing capacity, which overcomes the penetration of corrosive Cl^-^ ions through the pores and cracks, thereby improving the corrosion resistance of the mild steel surface in saline environment. The synergistic effect of Ch and GA combination in the nanocomposite can be ascribed to strong interaction of the empty d-orbital of the substrate metal atoms with the unshared electron rich polar sites in the heteroatoms (i.e., nitrogen or oxygen from the heterocyclic moiety) and/or the functional groups, like the hydroxyl or carboxylic functional group in the Ch/GA molecular structure ^27^. This can perfectly hinder the charge transfer between local anodic and cathodic sites, and thus restrict the steel corrosion.

**Table 2:** Polarization corrosion parameters and the protection efficiencies for the bare and different coated mild steel samples in 3.5 wt.% NaCl solution at 25 °C.

CGAC dose in the coating	*E*_corr_ (mV)	*i*_corr_ (µA cm^-2^)	*β*_a_ (mV/dec)	*β*_c_ (mV/dec)	*ɳ*_Tafel_ (%)	^*a*^SD
Bare steel	− 767	53.2	109	− 207	–	–
25 ppm	− 780	10.9	99.5	− 138	79.51	2
50 ppm	− 793	7.8	199	− 131	85.34	2.4
100 ppm	− 803	4.8	211	− 126	90.98	2.3
200 ppm	− 810	2.1	257	− 126	96.05	2.1

^*a*^SD, represents the standard deviation of three measurements.

#### Electrochemical impedance measurements

**Figure 10:**
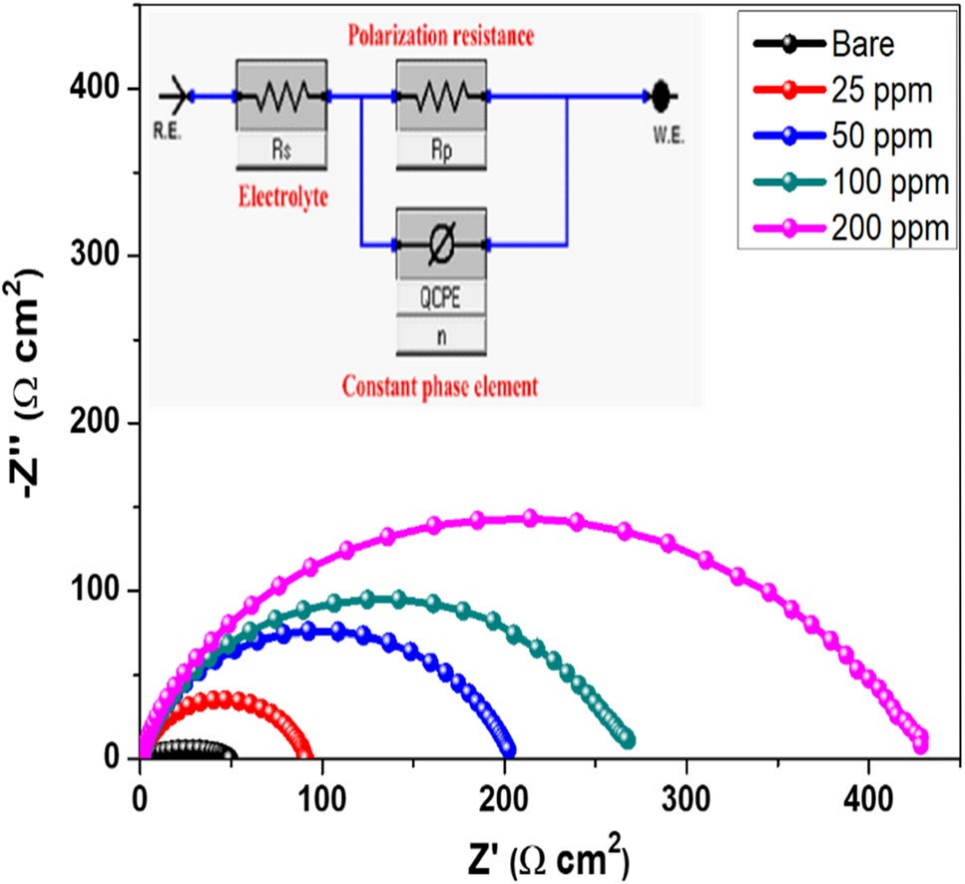
Nyquist plots of the bare and different coated mild steel samples in 3.5 wt.% NaCl solution at 25 °C (Inset: Equivalent circuit used to fit the experimental EIS data).

**Figure 11:**
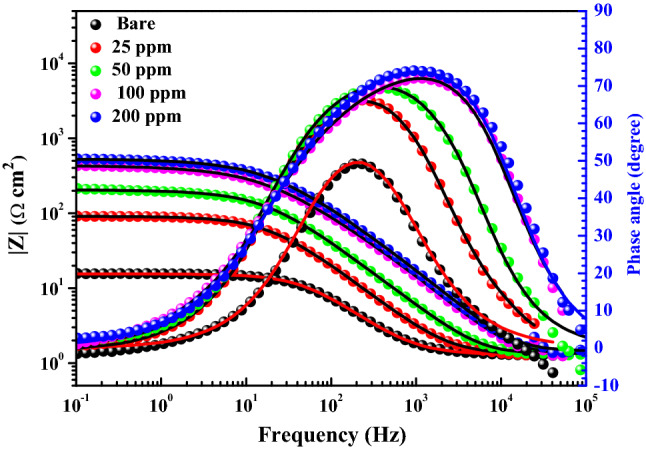
Bode plots of the bare and different coated mild steel samples in 3.5 wt.% NaCl solution at 25 °C.

EIS is an effective nondestructive technique utilized in this set of measurements to glean more important information about the protective behavior of Ch/GA nanocomposite coatings on mild steel in saline solution. EIS has the advantage that alongside the coat resistance value, the double layer capacitance value can also be obtained in the same measurement ^19^. Figures 10 and 11 represent the Nyquist and Bode plots of the impedance spectra for uncoated and different coated mild steel samples measured at the open-circuit potential (or the free *E*_corr_ potential value) after 1 h exposure in saline solution at 25 °C. Regarding the Nyquist format (Fig. 10), the spectra of tested steel samples exhibit one distinct capacitive loop at the high and medium frequencies associated with a single capacitive time constant on the Bode format (Fig. 11). The similar profile of the impedance spectra on the Nyquist and Bode plots indicates a similar corrosion mechanism for both the bare and coated mild steel samples and suggests an activation-controlled mechanism for its corrosion in saline media ^35,36^. It is also noticed upsurge in the semicircle diameter on the Nyquist plots, as well as a gradual rise in the absolute impedance (|*Z*|) and the phase maximum (*θ*_max_) values on the Bode plots with increasing the dose of CGAC nanopowder in the coated layer. These features indicate formation of more resistive layer on the steel surface that is further confirmed by the concomitant shift in the value of *θ*_max_ toward a lower frequency with increasing the CGAC powder dose in the coating. Such a behavior indicates a subsequent increase in the barrier properties of the coated layer as measured by the protection coefficient ($\eta_{R}$%) calculated using the following expression ^50,51^:

4 $$\eta_{R} \% \, = \left( {{1 } - \frac{{R_{p}^{b} }}{{R_{p}^{a} }}} \right) \times 100$$

where $R_{p}^{b}$ and $R_{p}^{a}$ are the polarization resistance values for both uncoated and coated mild steel electrodes, respectively. The broadness and increased value of the phase angle at its peak for coated samples with the powder dose indicate that the coated surface becomes smoother and thus more effective as a rigors barrier highly proficient in mitigating the degradation rate of mild steel in the saline media. A suitable electrical equivalent circuit (EC) is used to replicate the collected experimental EIS data and evaluate the impedance parameters. It is quite obvious that, capacitive loops on the Nyquist representation do not have a perfect half-circle shape. As reported previously, this behavior is related to capacitance dispersion at the interface ^52^, which can be measured in terms of a distributed electrical constant phase element (CPE). The impedance function of the CPE (*Z*_CPE_) is described by the following formula ^53^:

5 $$Z_{{{\text{CPE}}}} = Q^{{ - {1}}} (j\omega)^{ - n}$$

where the admittance *Q* and exponent* n* of the CPE are both independent of frequency (*f*), and all provide information regarding the degree of surface inhomogeneity. Deviation of the factor *n* from unity is an indication of deviation of *Q* (in Ω^-1^ cm^-2^ s^n^) from the real capacitance (in F cm^-2^).

Nevertheless, *Q* value obtained from the fitting for the bare and coated mild steel samples are identical to the double layer capacitance (*C*_dl_) values at *ω* = 1, where *ω* (in rad s^-1^) is the angular frequency (*ω* = *2* $\pi$ *f*, *f* being the signal frequency in Hz or s^-1^) ^54^. Accordingly, the EC model used in fitting the experimental EIS data (inset of Fig. 10) includes the single time constant (*R*_p_*Q*) parallel combination in series with the solution resistance (*R*_s_), and the resultant impedance parameters from the analysis procedure are all compiled in Table 3. As can be seen, the polarization resistance (*R*_p_) that implicitly involving the charge transfer, film, and pore resistances, shows a significant increasing trend in its value from uncoated mild steel to Ch/GA nanocomposite coated samples. Where, for the bare surface, it is 18.2 Ω cm^2^ and increases to 420.3 Ω cm^2^ for 200 ppm coated sample, which is about 25 times higher. Coating of a protective thin layer on the metal/solution interface is responsible for the upsurge in *R*_p_ value. In the meantime, *C*_dl_ has an opposite trend where it decreases with increasing the amount of CGAC nanopowder in the coating. Commonly, the double layer of the metal/solution interface can be demonstrated by the Helmholtz parallel plate capacitor model with a capacitance (*C*_dl_ or admittance *Q*) that is inversely related to thickness (*d*) of the coated layer on the steel sample through the expression ^55,56^:

5 $$C_{{{\text{dl}}}} = \varepsilon_{{\text{o}}} \varepsilon_{{\text{r}}} A/d$$

**Table 3:** Electrochemical impedance parameters and the corresponding protection efficiency (*ɳ*_R_%) for the bare and different CGAC coated steel samples in 3.5 wt.% NaCl solution at 25 °C.

CGAC dose in the coating	*R*_s_ (Ω cm^2^)	*C*_dl_ (µF cm^-2^)	*n*	*R*_p_ (Ω cm^2^)	*ɳ*_R_ (%)
Bare steel	1.32 ± 0.5	153 ± 0.6	0.89	18.2 ± 0.3	–
25 ppm	1.24 ± 0.1	82 ± 0.4	0.86	89.7 ± 0.7	79.71
50 ppm	1.28 ± 0.3	79 ± 0.3	0.88	130.5 ± 0.2	86.05
100 ppm	1.06 ± 0.1	52 ± 0.5	0.85	190.7 ± 0.4	90.46
200 ppm	1.25 ± 0.2	16 ± 0.1	0.87	420.3 ± 0.2	95.67

where *ε*_o_ is the permittivity of free space (8.854 × 10^–14^ F cm^−1^), *ε*_r_ is the relative dielectric constant of the coated film, and *A* is the geometric surface area (cm^2^) of the surface. In reality, a decrease in *C*_dl_ values can be caused by an increase in the thickness of the electrical double layer and/or a decrease in the local dielectric constant. Accordingly, the steady replacement of water molecules and other ions adsorbed originally on the steel surface by inhibitor composite molecules in the coating is thought to be the cause of the drop in *C*_dl_ value ^57^. The increase in *R*_p_ and decrease in *C*_dl_ with further increase in the CGAC nanopowder in the coat suggests that Ch/GA nanocomposite film works as a major interface barrier layer where its polarization resistance regulates mild steel corrosion at the open circuit settings. Ch/GA nanocomposite placed at the mild steel/chloride solution interface form a thin layer of coating on the steel surface that actively blocks corrosive sites and considerably improves the coating barrier characteristics. This film at the interface hinders additional anodic and cathodic reactions resulting in higher *R*_p_ values ^58^. The increased protection efficiency identified from the Tafel polarization method is also shown from the present impedance approach. This adds to the evidence that designed Ch/GA nanocomposite coatings significantly improve the corrosion resistance of the mild steel sample in saline solution.

It is to be noted that, on the Bode format (Fig. 11) the absolute impedance (|*Z*|) for all coated samples is always much higher than for the bare substrate, and that its value increases as the film thickness is increased with increasing the CGAC nanopowder in the coating. This suggests that in presence of Ch/GA nanocomposite films, the rate of corrosion is reduced and continues to decrease when the film thickness is increased. An increase in the inhibitor concentration would result in a thicker protective layer formed on the mild steel surface, preventing the electrochemical corrosion. It can be seen that protection efficiency increases by increasing the amount of Ch/GA nanocomposite powder in the coating, recording 95.67% protection efficiency (${\eta }_{R}\%$) at a dose of 200 ppm-CGAC nanocomposite coating. Nanocomposites Ch/GA coatings have the potential to suppress corrosion by acting as a physical barrier of a passive layer on the mild steel surface, inhibiting corrosion by reducing the passage of aggressive ions through the coating and thus impeding the charge transfer between local anodic and cathodic sites as well as the fine dispersion of electrical conductivity within the polymeric matrix.

#### Effect of immersion time

**Figure 12:**
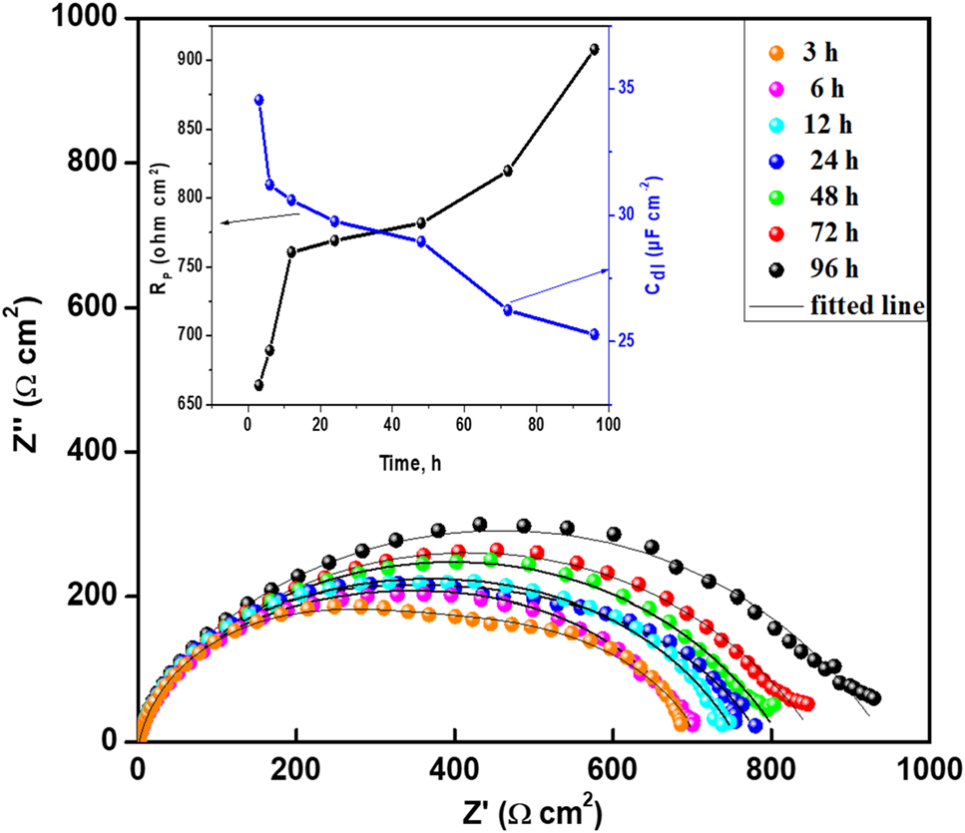
Time dependence of the impedance spectra of 200 ppm CGAC coated mild steel sample in 3.5 wt.% NaCl solution at 25 °C. Inset: Variation of *R*_p_ and *C*_dl_ values with the immersion time.

Electrochemical impedance spectroscopy is an excellent approach for quantification of long-term coating performance since it does not cause any considerable system disturbance^54,59^. Therefore, in this set of measurements, EIS was exploited to give a quick assessment for the predictable stability and durability of nano-CGAC coating over an extending period of time. Figure 12 displays the Nyquist diagrams of mild steel coated by 200 ppm Ch/GA nanocomposite, recorded after different immersion periods of 3, 6, 12, 24, 48, 72, and 96 h in 3.5 wt.% NaCl solution. As can be clearly seen, there is a progressive increase in the loop size of the impedance spectra with protracting the time. Indeed, the immersion time can favour the interaction between the functional groups in the nanocomposite molecular structure and the empty orbitals of the metal atoms in the surface. This would lead to an effective sealing of the steel surface as the time is prolonged, indicating sustainable coating. The variability of *R*_p_ and *C*_dl_ with immersion time is depicted in detail in the inset of Fig. 12. Plainly, the results show that polarization resistance (*R*_p_) value increases significantly from 664 to 907 Ω cm^2^, with a simultaneous dramatic lowering in the double layer capacitance value from 34.5 to 25.2 µF cm^-2^. In addition, there was no any visible degradation sign on the coating after 96 h exposure to the aggressive saline solution.

#### Contact angle

**Figure 13:**
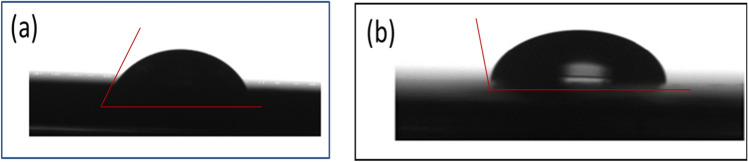
Water droplet contact angle on mild steel surfaces exposed 24 h to 3.5 wt.% NaCl solution: (**a**) bare surface, and (**b**) coated surface by Ch/GA nanocomposite.

Contact angle measurements are one of the accessible and more relevant criteria for studying the development of thin coatings on steel surfaces. To further verify the development of Ch/GA nanocomposite layer, water contact angle measurements were executed on a mild steel surface. After 24 h exposure to 3.5 wt.% NaCl solution, the water droplet contact angle was measured on (a) bare mild steel surface, and on (b) coated mild steel surface with Ch/GA nanocomposite thin film. Upon exposure to the saline solution, the contact angle on the uncoated bare surface was found to be so acute (59.7°) due to the high affinity of mild steel surface to water molecules and the sever corroded surface by the aggressive media, as well as the strong hydrophilicity of the corrosion products. In the meantime, Ch/GA nanocomposite film coating on the steel surface greatly improves the ability of mild steel to resist water molecule adhesion to its surface. Experimentally Fig. 13 shows that the water droplet contact angle increases from a small acute value of 59.7° for the bare surface to a large obtuse angle value of 101.2° for the coated sample. This detection clearly demonstrates that in saline solution the surface film of Ch/GA nanocomposite coating is smoother and has more strengthen hydrophobic property as compared to the bare corroded mild steel surface^60^.

#### Surface morphology tests

**Figure 14:**
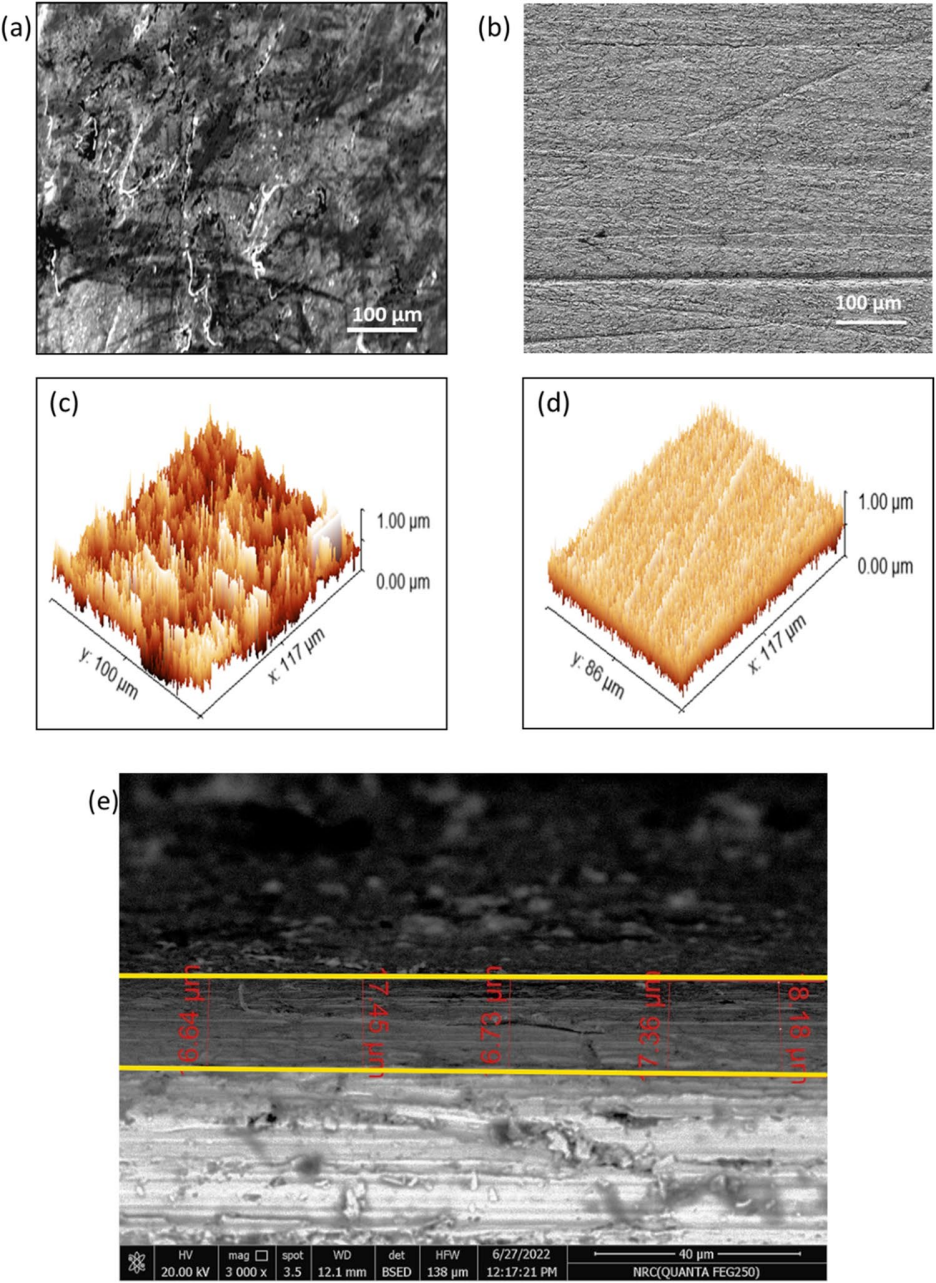
SEM micrographs for: (**a**) bare and (**b**) mild steel coated by Ch/GA nanocomposite. The 3D AFM images for (**c**) bare and (**d**) mild steel coated by Ch/GA nanocomposite. (**e**) The SEM cross-sectional image of mild steel coated by Ch/GA nanocomposite.

After being submersed for 24 h in 3.5 wt.% NaCl solution, SEM micrographs were recorded for both bare sample and Ch/GA nanocomposite coated mild steel sample surfaces. In the absence of CGAC coating, Fig. 14a reveals the morphology of bare mild steel surface as a very rough and extensively corroded surface. However, compared to the morphology of the uncoated sample surface, the Ch/GA nanocomposite coating on mild steel surface can serve as a barrier against the ingress of chloride ions from the medium toward the substrate, subsequently hindering the metal corrosion and avoiding the damage in its surface as shown in Fig. 14b. By converting SEM into three-dimensional surface topographical characteristics, the 3D AFM images for the bare mild steel sample (Fig. 14c), and the mild steel coated by Ch/GA nanocomposite (Fig. 14d) were measured and inspected after 24 h immersion in the saline medium. Compared to the bare steel surface, it can be seen that coated steel sample characterizes by a rise up in the thickness of its surface film due to the placement of the coated layer. Also, the mean roughness (*R*_a_) value was found to be greatly reduced from 220 to 111 nm only after applying Ch/GA nanocomposite coating on the sample surface. The reason can be traced to the compactness and smoothness in the surface morphology of the top layer for the coated sample in good agreement with the contact angle results. It is also obvious from the cross-sectional view shown in Fig. 14e that, mild steel coated by Ch/GA nanocomposite has an average thickness of 17 µm and the coating layer well adheres to the substrate surface.

## Conclusions

Eco-friendly and sustainable Ch/GA composite (CGAC) crystalline nanopowder was successfully synthesized with a facile ultrasonic-assisted route for anticorrosion application on mild steel in neutral saline media. The acquired results from gravimetric and electrochemical testing methods revealed good protection efficiency that was enhanced with the upsurge of CGAC dose in the coated layer achieving 96.9% for the 200 ppm-coating. Tafel polarization plots clearly indicated that the protection capability of CGAC nanopowders in the coated layers behaved as mixed adsorbed inhibitors, which helped to block both active anodic and cathodic corrosion sites on the steel surface. Moreover, EIS data revealed a significant increase in the surface film resistance and a parallel decrease in its capacitance value with increasing the CGAC dose in the coated layer. This was attributed to the increase in the surface film thickness with the increase in the amount of CGAC nanopowders. SEM micrographs and 3D AFM roughness images for uncoated and coated steel samples after exposure in the corrosive media presented further support to the obtained experimental findings. In line with that, the water droplet contact angle was also found to increase from a small acute value of 59.7° for the bare uncoated steel surface to a large obtuse value of 101.2° for the coated one.

The findings of this current research provide an insight into the anticorrosion capability of novel green coatings based on Ch/GA nanocomposite. The effective improvement in the corrosion resistance of the steel sample utilizing Ch/GA nanocomposite coatings as surface barriers is considered a recent trend in the corrosion mitigation field for the marine environment.

## Data Availability

Correspondence and requests for materials should be addressed to F.E.T.H. All data generated or analysed during this study are included in this article.
