# Author Correction: Green and sustainable chitosan–gum Arabic nanocomposites as efficient anticorrosive coatings for mild steel in saline media

**DOI:** 10.1038/s41598-022-21477-w

**Published:** 2022-10-06

**Authors:** Sherief A. Al Kiey, Mohamed S. Hasanin, Fakiha El-Taib Heakal

**Affiliations:** 1 Electrochemistry and Corrosion Department, National Research Centre (NRC), Dokki, 12622 Cairo, Egypt; 2 Cellulose and Paper Department, National Research Centre (NRC), Dokki, 12622 Cairo, Egypt; 3 Chemistry Department, Faculty of Science, Cairo University, Giza, 12613 Egypt

Correction to: *Scientific Reports* https://doi.org/10.1038/s41598-022-17386-7, published online 01 August 2022

In the original version of this Article, Mohamed S. Hasanin was omitted as a corresponding author. Correspondence and requests for materials should also be addressed to sido_sci@yahoo.com.

The original Article has been corrected.

